# Effectiveness of a Multi-component Delirium Prevention Program Implemented on General Medicine Hospital Units: an Interrupted Time Series Analysis

**DOI:** 10.1007/s11606-023-08238-9

**Published:** 2023-07-10

**Authors:** Judith Versloot, Simona C. Minotti, Samia Amer, Amna Ali, Julia Ma, Mary-Lynn Peters, Hana Saab, Terence Tang, Jason Kerr, Robert Reid

**Affiliations:** 1 Institute for Better Health, Trillium Health Partners, Mississauga, ON Canada; 2 Institute of Health Policy, Management & Evaluation, University of Toronto, Toronto, ON Canada; 3 Department of Statistics and Quantitative Methods, University of Milano-Bicocca, Milan, Italy; 4 Trillium Health Partners, Mississauga, ON Canada; 5 Department of Medicine, University of Toronto, Toronto, ON Canada

**Keywords:** delirium prevention, delirium program, inpatients, internal medicine

## Abstract

**Background:**

Delirium is among the most prevalent harmful events in hospitals that is associated with an elevated risk for severe outcomes such as functional decline, falls, longer length of stay, and increased mortality.

**Objective:**

To evaluate the impact of the implementation of a multi-component delirium program on the prevalence of delirium and the incidence of falls among patients staying on general medicine inpatient hospital units.

**Design:**

A pre-post intervention study using retrospective chart abstraction and interrupted time series analysis.

**Cohort:**

Patients were selected from adult patients that stayed at least 1 day on one of the five general medicine units in a large community hospital in Ontario, Canada. A total of 16 random samples of 50 patients per month for 8 consecutive months pre-intervention (October 2017 to May 2018) and 8 months post intervention (January 2019 to August 2019) were selected for a total of 800 patients. There were no exclusion criteria.

**Intervention:**

The delirium program included multiple components: education of staff and hospital leadership, twice per day bed-side screen for delirium, non-pharmacological and pharmacological prevention, and intervention strategies and a delirium consultation team.

**Measurement:**

Delirium prevalence was assessed using the evidence-based delirium chart abstraction method, CHART-del. Demographic data as well as fall incidence were also collected.

**Result:**

Our evaluation showed that the implementation of a multicomponent delirium program led to a reduction in delirium prevalence and fall incidences. The reduction in both delirium and falls was the largest for patients in the ages between 72 and 83 years old and varied across inpatient units.

**Conclusion:**

A multi-component delirium program to improve the prevention, recognition, and management of delirium reduces the prevalence of delirium and fall incidence among patients in general medicine units.

**Supplementary Information:**

The online version contains supplementary material available at 10.1007/s11606-023-08238-9.

## INTRODUCTION

### Problem Description

Delirium is a common multifactorial condition among hospitalized patients that is associated with an elevated risk for severe outcomes such as functional decline, falls, longer length of stay, and increased mortality.^1–3^ It is also very common, accounting for 10% of all reported harmful hospital events in Canada,^4^ especially in older patients where delirium prevalence is estimated to be as high as 20%. Despite that up to 30–40% of cases is preventable^5^, it is often not recognized by clinicians in the early stages ^6–10^, and efforts to reduce the incidence of delirium are challenged by lack of delirium awareness; the conflation of delirium with dementia; and the absence of training and in-service education programs for physicians, nursing, and allied health staff.^8,9^

### Available Knowledge

To address the multiple factors affecting the prevention and care for patients with delirium, multicomponent interventions are needed ^11^ and have been shown to reduce delirium incidence from 3% up to 30% in randomized trials as compared to usual care, ^12–14^ thereby improving the quality of life for patients and their families, while reducing healthcare costs and societal burden of delirium.^15^. These interventions often include the use of education, implementation of both non-pharmacological and pharmacological prevention strategies, and the support of inter-professional teams at the point of care to address potentially modifiable risk factors.^15–17^

### Rationale

Our local hospital did not have a formalized delirium program. Therefore, based on the available knowledge, we developed, implemented, and evaluated a practical, evidence-informed multicomponent program for the prevention, early recognition, and effective management of delirium at a large community hospital.

### Specific Aims

In this paper, we present the evaluation of a multi-component delirium program in a large community hospital serving a diverse population to understand the effectiveness of the intervention in reducing the number of delirium cases and falls analysed with pre-post Interrupted Time Series (ITS) design.

## METHODS

### Context

The delirium program was implemented at Trillium Health Partners (THP), a large community hospital, in Mississauga, Canada, serving a diverse population of over one million people. With 1379 inpatient beds, the hospital provides the full range of acute care hospital services. In the 5 years leading up to this implementation, a few other initiatives related to delirium and falls were initiated at THP. First, the organization attained Registered Nurses Association of Ontario (RNAO) Best Practice Spotlight Organization (BPSO) certification (2012–2015), during which time the Prevention of Falls and Fall Injuries in Older Adults Best Practice Guideline (BPG) was implemented across multiple units. After this, the RNAO Delirium, Dementia and Depression in Older Adults: Assessment and Care BPG was implemented in conjunction with the delirium program activities (2015–2021). This BPG was intentionally launched concurrently with the work related to the delirium program. The BPG team supported the educational efforts for the delirium program. The journey to BPSO certification could have stimulated a culture of change and learning which could have had a positive influence on the implementation and uptake of the delirium program.

This evaluation studied charts from patients that stayed on one of the 5 general medicine units at one of the three hospital sites. Implementation of the delirium program in these units started in June 2018. This evaluation examines the occurrence of study outcomes within the pre-implementation period (October 2017–May 2018) and the post-intervention period (January 2019–August 2019). This manuscript follows the Revised Standards for Quality Improvement Reporting Excellence (SQUIRE) reporting guidelines.^18^

### Multicomponent Delirium Intervention

To change the existing model of delirium care in the organization, a team of administrators, clinicians, and scientists formed a project team and developed a multi-component delirium program based on current evidence. The developed delirium program intends to achieve three goals: (a) to prevent the onset of delirium, (b) to identify delirium when present, and (c) to improve the evidence-based management of delirium when identified.

Before implementation of the delirium program on inpatient hospital units, the project team engaged with “local” leadership (program directors, medical directors, unit managers, and charge nurse/clinical leaders) and staff to tailor the program to the context of the units. The pre-implementation activities included, but were not limited to, current state mapping, recruitment and training of delirium champions on each unit, and the creation of a delirium data dashboard and screening rate score cards for the units.

**Figure 1:**
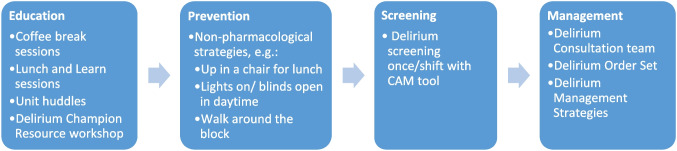
**Delirium program components.**

After the pre-implementation activities were completed, all clinical staff of the units were trained and the Delirium Program was implemented on the hospital units. The key components of the delirium program are the following: education on delirium for all staff involved, non-pharmacological and pharmacological delirium prevention strategies, delirium recognition with a bedside screener, and specific delirium management processes (order sheet) and strategies (see Fig. 1). More details can be found in Appendix 1.

### Evaluation Study of a Multi-component Delirium Intervention

#### Implementation Assessment

**Figure 2:**
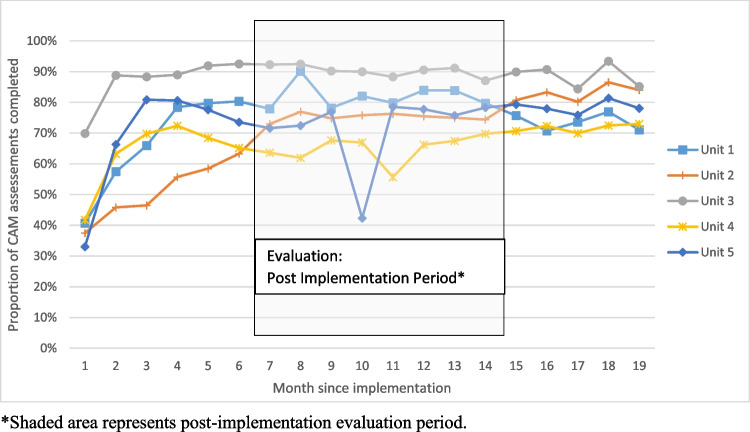
**Implementation of twice-daily CAM screen on the General Internal Medicine Units.**

In order to assess the implementation success of the delirium program, the two daily bedside delirium screening rates were calculated. The percentage of delirium screens done monthly for each of the 5 units was computed by dividing “the total number of delirium screens performed monthly per unit” over “the number of monthly bed days per unit, times 2” (times 2 since the delirium screen is done twice per day). The tracking of the delirium screening was done for 19 months after the start of the intervention for all patients that stayed on a unit in each given month to assess the implementation success; see Fig. 2. The post intervention chart abstraction was done for selected patients that stayed on the units in months 7–14 after the start of the implementation (see further the section below).

#### Effectiveness

Previous evaluations of multicomponent interventions about delirium often use post-intervention study designs with small sample size, thereby limiting the assessment of effectiveness and generalizability.^15^ In order to assess the effectiveness of the multicomponent delirium program (hereafter also referred to as the intervention), we applied a pre-post evaluation study design using retrospective chart abstraction and Interrupted Time Series (ITS). ITS has increasingly been advocated as one of the more robust observational quasi-experimental designs for the evaluation of health system quality improvement interventions. The ITS design relies on data collected at multiple intervals over time before and after an intervention to establish a causal relationship between an intervention (e.g., delirium program) and an outcome of interest (e.g., delirium or falls).^19^ Based on Penfold and Zhang’s (2013), for our analysis, we considered a time period of 8 months before and after implementation of the intervention.^20^ For each month, we randomly selected a sample of 50 patients that stayed on any one of the 5 general medicine units. Patients were selected based on the month they were admitted to the hospital. For the *pre-implementation period*—October 2017 to May 2018—we selected 50 patients per month from 8 consecutive months in the period immediately prior to implementation of the delirium program in June 2018 (*n* = 400 patients). For the *post-implementation period—*January 2019 to August 2019—we selected 50 patients per month from 8 consecutive months starting 7 months after implementation of the delirium program to allow for the intervention to be fully implemented into practice (*n* = 400 patients). No stratification for unit or any other variable was used.

### Patient Cohort

The study population included 800 adult patients aged 18 years or older who stayed for a minimum of 1 day on one of the 5 general medicine units during the data collection period (pre- or post-intervention). There were no exclusion criteria. The patient records covering their entire stay, including time in the emergency room and on other units, were used.

### Measures

#### Delirium Screening

The bedside delirium screening was done using the Confusion Assessment Method (CAM)^21–23^, a brief delirium diagnostic tool that is accurate (sensitivity 86%, specificity 93%), with high inter-observer reliability.^24^

#### Delirium: CHART-del

The presence of delirium was assessed based on either clear physician-diagnosis of delirium as documented in physicians’ progress notes, consults or discharge summary, or by the validated chart-based identification method CHART-del.^25^ The chart abstraction involved a detailed review of the full patient’s medical records (a combination of paper and electronic medical records) and was designed with the goal of maximizing sensitivity for identification of delirium.^25^ Information on acute changes in mental status, time and duration of such episodes, evidence of agitation and reversibility or improvement of the acute confusion, and scanning and auditing medical records for the key words/terms for delirium identification were abstracted. Appendix 2 describes the data collection tool (CHART-del tool). The CHART-del tool is a validated instrument with good sensitivity and specificity (74% and 83% respectively) when compared to clinical assessment.^25^

#### Specialist consultation, restraint or safety attendant, falls, and bedside delirium screen

The following outcomes were collected for each patient during their entire hospital stay from their medical chart: received a specialist consultation, use of restraint, safety attendant/sitter, had a fall, and whether or not a screening was done for delirium using the CAM ^22,23^.

Data collection was performed by a research assistant (SA) who was fully trained in the CHART-del evidence-based method. Calibration and training had taken place as part of one other large study using the same methods.

### Statistical Analysis

Descriptive statistics were performed, and values are presented as means and standard deviations (SD) for continuous values and counts (%) for categorical variables. Segmented regression was used to assess the impact of the multicomponent delirium intervention on the number of delirium cases and falls, by estimating the changes in level (and trend) in the post-intervention period compared to the pre-intervention period. Segmented regression analysis is a statistical method for modelling the interrupted time series data to draw more formal conclusions about the impact of an intervention on the measure of interest ^26^ (see Appendix 3). The datasets used and/or analyzed during the current study are available from the corresponding author on request.

### Ethical Considerations

The THP Research Ethics Board approved this evaluation. Patients did not consent since the study operated under a waiver of patient consent because of the low risk of chart abstraction methodology.

## RESULTS

### Cohort

The characteristics of the patients in the pre- and post-intervention cohorts are reported in Table 1. There were no differences in the distribution of sex, age, place of residence, or hospital admission route between the pre- and post-intervention cohorts.

**Table 1:** Patient Cohort Demographics

	Pre-intervention *N* = 400	Post intervention *N* = 400
Sex (*n* (%))		
Female	217 (54.4%)	200 (51.1%)
Age on admission (mean (SD))	69.1 (17.7)	67.5 (18.8)
Age groups (*n* (%))		
18–57 58–71 72–83 > 83	98 (24.5%) 92 (23%) 119 (29.8%) 91 (22.8)	112 (28%) 104 (26%) 96 (24%) 88 (22%)
Place of residence (*n* (%))		
Home independently Home with support services Retirement home (little to no help with everyday tasks) Nursing home (substantial or complete assistance with everyday tasks) Other	262 (65.5%) 80 (20.0%) 25 (6.3%) 25 (6.3%) 8 (2.0%)	277 (69.3%) 74 (18.5%) 21 (5.3%) 22 (5.5%) 6 (1.5%)
Hospital admission route (*n* (%))		
Emergency Department Planned admission to unit Referral from outpatient service Hospital transfer Other	369 (92.3%) 19 (4.8%) 3 (0.8%) 6 (1.5%) 3 (0.8%)	353 (88.3%) 25 (6.3%) 10 (2.5%) 11 (2.8%) 1 (0.3%)

### Implementation Assessment

As a measure of the implementation success of the delirium program, the two daily CAM screenings were tracked for the medicine units. Figure 2 represents the uptake for the first 19 months after implementation. The period in the post-implementation phase that is used for the evaluation is shaded in gray. The average CAM screening rates over our study period ranged from 65% for unit 4, 72% for unit 5, 75% for unit 2, 82% for unit 1, to 90% for unit 3.

### Effectiveness

#### Main Results

**Figure 3:**
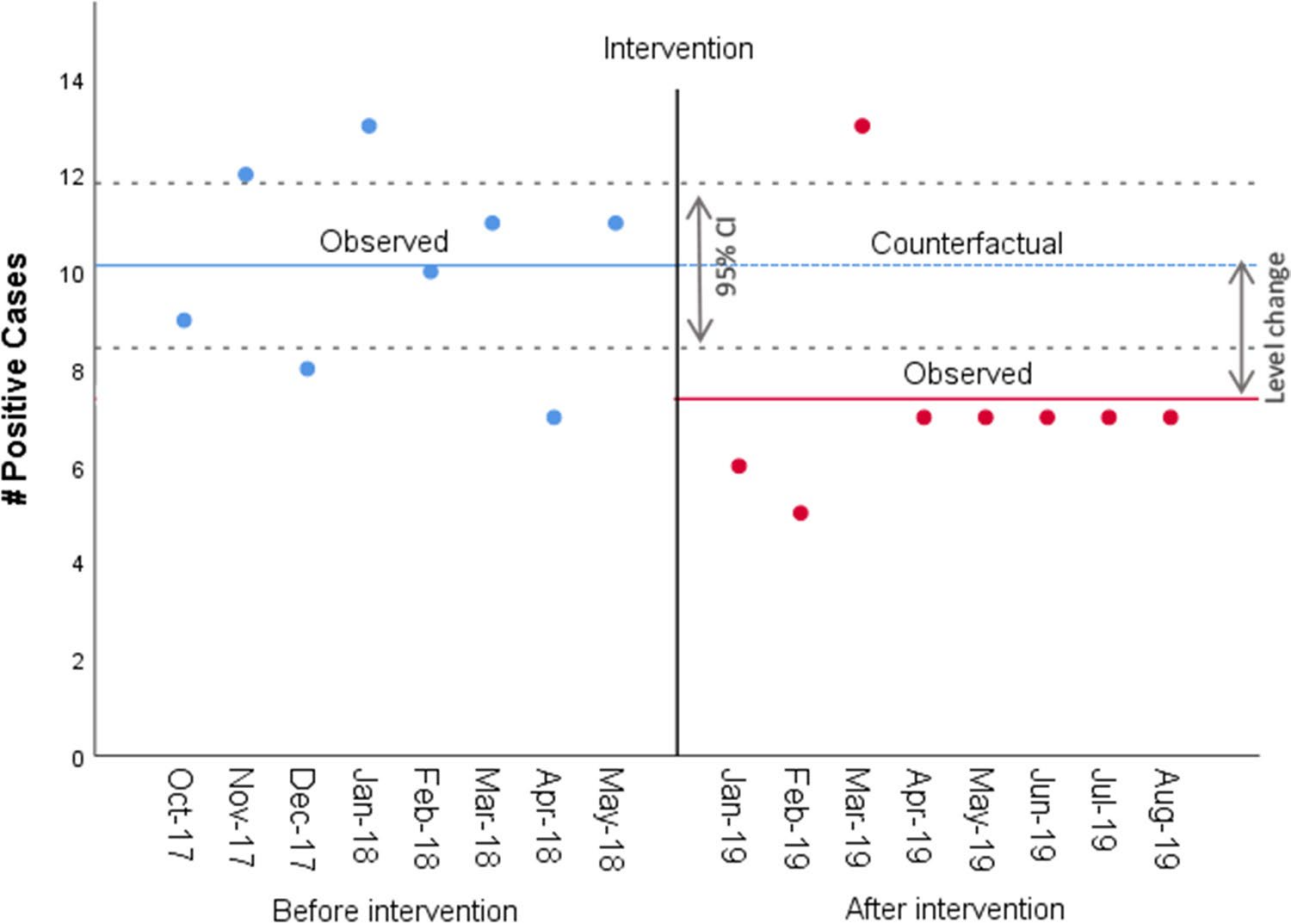
**Graphical representation of the Interrupted Time Series for counts of patients with delirium.**

The average number of patients with delirium per month over 50 patients decreased significantly from 10.25 (SD 1.83) before the implementation of the intervention to 7.38 (SD 2.39) after (see Table 2, Fig. 3, and Appendix 3) (*p* = 0.026, Std. error = 1.11). This is a relative reduction of 27.15% in the number of delirium cases per month. The estimate of the intervention effect did not change after adjusting the linear model for the negative autocorrelation in the error term, whereas it slightly reduced after accounting for the heterogeneity across units but remained significant (*p* = 0.037, Std. error = 1.021) (see Appendix 3, Table 1a).

**Table 2:** Parameter Estimates, Standard Errors, and *P*-Values from the Most Parsimonious Segmented Regression Models Predicting Monthly Number of Delirium Cases and Falls

Delirium	Coefficient	Standard error	*t*-statistic	*P*-value
Intercept	10.13	0.78	12.92	0.00
Level change after intervention	–2.75	1.11	–2.48	0.03
Falls	Coefficient	Standard error	*t*-statistic	*P*-value
Intercept	14.5	0.9	16.04	0.00
Level change after intervention	–2.75	1.28	–2.15	0.05

**Figure 4:**
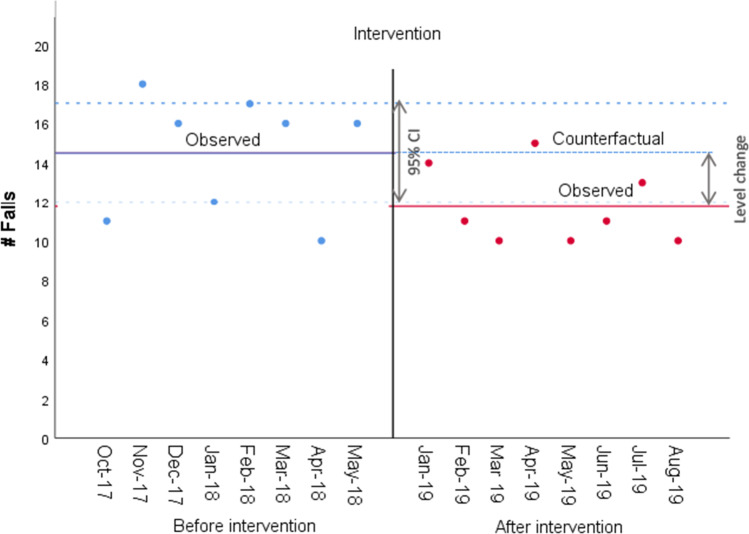
**Graphical representation of the Interrupted Time Series for counts of patients with falls.**

The average number of patients who experienced a fall per month over 50 patients decreased significantly from 14.5 (SD 3.0) before the implementation of the intervention to 11.8 (SD 2.0) after (see Table 2, Fig. 4, and Appendix 3) (*p* = 0.049, Std. error = 1.28). This is a relative reduction of 18.97% in the number of falls per month. The estimate of the intervention effect did not change after adjusting the linear model for the negative autocorrelation in the error term, whereas the intervention effect reduced after accounting for the heterogeneity across units and became non-significant (*p* = 0.538, Std. error = 1.271) (see Appendix 3).

#### Explorative Analysis

As an explorative analysis, we examined the distribution of the number of delirium cases and falls within different age groups and within individual hospital units to gain insights into which segment of patients benefited most from the intervention. These results showed a 14.7% reduction in delirium among patients aged between 72 and 83 years of age (relative reduction 65.4%) (*p* = 0.01) and a 21% reduction in delirium among patients that stayed on Unit 3 (relative reduction 59.5%) (*p* = 0.03). Furthermore, we saw a 10.3% reduction in falls among patients that stayed on Unit 1 (relative reduction 41.2%) (*p* = 0.03). Although most other age groups and units saw a decrease in delirium and falls, none of the differences reached statistical significance, for some instances likely due to lack of power, since the study was not designed for this kind of explorative analysis, or due to a floor effect. Also of note is the large spread in delirium rates, from 10 to 35%, and falls, from 10 to 23%. Additionally, there is a large spread from pre-intervention to post-intervention in the absolute difference in delirium across the units: from an increase of 2% to a decrease of 21%; and for falls: from an increase of 2.5% to a decrease of 13.3% for falls (see Tables 3 and 4).

**Table 3:** Distribution of Delirium Among Different Age Groups and Different Hospital Units

		Pre-intervention Patients with delirium		Post intervention Patients with delirium	***P-value***
	***N***	***n (%)***	***N***	***n (%)***	
All patients	400	82 (20.5%)	400	59 (14.8%)	0.03
Age:					
18–57 58–71 72–83 > 83	98 92 119 91	6 (6.1%) 9 (9.8%) 32 (26.9%) 35 (38.5%)	112 104 96 88	8 (7.1%) 7 (6.7%) 9 (9.4%) 35 (39.8%)	0.77 0.44 0.01 0.86
Hospital unit					
Unit 1 Unit 2 Unit 3 Unit 4 Unit 5	133 58 34 90 84	17 (12.8%) 6 (10.3%) 12 (35.3%) 23 (25.6%) 23 (27.4%)	136 65 49 73 77	14 (10.3%) 8 (12.3%) 7 (14.3%) 15 (20.5%) 15 (19.5)	0.52 0.73 0.03 0.45 0.24

**Table 4:** Distribution of Falls Among Different Age Groups and Different Hospital Units

		Pre-intervention		Post-intervention	***P-value***
	***N***	***n (%)***	***N***	***n (%)***	
All patients	400	116 (29.0%)	400	94 (23.5%)	0.08
Age					
18–57 58–71 72–83 > 83	98 92 118 91	13 (13.3%) 17 (18.5%) 42 (35.6%) 44 (48.4%)	112 104 96 87	14 (12.5%) 17 (16.3%) 24 (25.0%) 39 (44.8%)	0.87 0.69 0.10 0.64
Hospital unit					
Unit 1 Unit 2 Unit 3 Unit 4 Unit 5	133 58 34 90 83	33 (25.0%) 17 (29.3%) 13 (38.2%) 25 (27.8%) 27 (32.1%)	136 65 49 73 77	20 (14.7%) 20 (30.8%) 13 (26.5%) 24 (33.3%) 17 (22.1%)	0.03 0.86 0.26 0.44 0.15

In Table 5, the descriptive statistics of the secondary outcomes before and after the implementation of the intervention are reported. The number of bedside screens for delirium was significantly higher after the implementation (97.8%) compared to before (20%), indicating an excellent uptake of this part of the intervention. Furthermore, no significant differences were found for length of stay, restraint, or safety attendant use.

**Table 5:** Secondary Outcomes for Pre- and Post-intervention Periods

	Pre-intervention *N* = 400	Post intervention *N* = 400	*P*-value
	*n* (%)	*n* (%)	
Bedside screen for delirium (at least 1 CAM screen)	80 (20.00%)	395 (98.75%)	*P* < 0.01
Restraint use	11 (2.75%)	10 (2.50%)	0.83
Safety attendant	2 (0.50%)	4 (1.00%)	0.41
	Mean (SD)	Mean (SD)	
Length of stay	9.3 (22.3)	9.4 (14.8)	0.95

## DISCUSSION

### Summary

Our evaluation of the multicomponent delirium program showed that the implementation led to a reduction in the number of patients with delirium, as well as a reduction in the number of patients that experienced a fall. The impact on falls should be interpreted with caution since the model that corrected for heterogeneity was not significant for the falls outcome. The reduction in delirium and falls was the largest in patients aged between 72 and 83 years old. Both older and younger patients were not improving as much. An individual’s delirium risk depends on both pre-disposing factors that influence ones’ vulnerability and precipitation factors that determine how noxious an insult is for the individual. The level of both of these determines one’s risk for delirium.^27^ In our case, it is likely that the older hospitalized adults aged 83 and above are very vulnerable so that even a mild insult can lead to the development of delirium thus making prevention challenging. On the contrary, in the younger population that is less vulnerable, only severe or repeated insults will lead to the development of delirium. These more severe insults such as surgery might not be preventable. A similar argument can be made for our fall results. Falls is a common geriatric syndrome that is multifactorial and mostly the result of a combined action of predisposing and precipitating factors much like delirium. Furthermore, falls and delirium share similar risk factors such as age and cognitive decline.^28^

In addition, our evaluation shows that the impact of the delirium program was not universal across all engaged hospital units. There are likely several factors at play that could potentially have influenced these results. First, we see that the uptake of the overall intervention, as approximated by the implementation of the bedside screen for delirium with the CAM tool, varied across different units. The unit (no. 3) with the best uptake of the bedside screen did have the largest reduction in delirium rates which could indicate that when the program is successfully implemented it has the potential to significantly reduce delirium rates. Screening for delirium, however, was only one of the core components of the delirium program; therefore, caution needs to be taken with the generalization to the implementation success of the full program. Second, we noticed that the baseline delirium rate had a large variation across units. For some units where this rate was relatively low compared to other units, we did not see much change after implementation of the program, which could have been the result of a floor effect. Third, implementing an elaborate program is labor intensive as it involves many healthcare professionals coordinating multiple tasks. Studies show that the “readiness” of hospital units and the unit context (time, workload, workflow, competing priorities, and staff turnover) are key factors to the success of new programs.^29^ Finally, as noted above, the risk for the development of delirium is multifactorial. A difference in any factor across units could have influenced the effectiveness of the delirium program on each particular unit. The statistical analysis applied in the current study, however, corrected for potential heterogeneity across the units and showed a significant impact of the multi-component delirium intervention on the reduction of delirium after controlling for heterogeneity.

This multi-component delirium program was well resourced with the dedication of project leads, a dedicated project team, and the luxury of time to try things out. It remains a challenge with multicomponent interventions to pinpoint which components were key to the success on some units and which components were lacking on the less successful units. Previous research has shown the importance of education, champions, screening, and continuous support from hospital management.^30^ For future research, it would be interesting to measure these in order to expand the knowledge on which preconditions and key components contribute to success (e.g., adequate staffing levels; strong leadership support; clinical and managerial engagement) using a mixed methods design.

In our study population, the implementation of the delirium program did not change the length of stay, the use of safety attendants, or restraints. The length of hospital stay depends on a wide variety of factors that are related to, for example, the setting, clinical care, or logistics. Although the delirium program targeted some components related to these factors such as clinical pathways,^31^ patient mobility,^32,33^ medication management^34^, and collaborative care, it is important to underline that it was not specifically designed to reduce length of stay.

The implementation of delirium prevention programs within hospitals is currently at the discretion of hospital leadership and as a result not universally applied. Therefore, the creation of a standard delirium strategy within all acute care centers that focuses on components such as prevention, screening, and delirium management strategies that are evidence based is needed. Currently, hospitals in Ontario need to report the incidence of delirium^35^ which is a good start to have hospitals incentivize systematic screening and improve awareness; however, without a mechanism to respond to a positive delirium screen, this effort will fall short in improving delirium-related morbidities and mortalities.

Unlike many other studies, no specific exclusion criteria were applied other than being hospitalized on a medicine unit. This allows for an increased generalizability of our findings to other hospitals’ medicine units. The use of chart abstraction in this study allowed data collection for both the pre-intervention period and post-intervention period providing data suitable for a strong evaluation design using ITS analysis. Other studies evaluating delirium prevention programs are often limited to data from the post-intervention period or report on other main outcomes than delirium. We do recognize that a limitation of the use of the chart abstraction method is that it is inevitable that some cases might be systematically mislabeled as either false negative or false positive. However, previous research demonstrated that the CHART-del method has a reasonable sensitivity and specificity and any under- or overestimation should be stable over time and temporal trends should remain meaningful.^25^ Another limitation of the study was the use of aggregate data for each time point (i.e., data were aggregated across units). Current ITS methods, including segmented regression, do not account for the heterogeneity among patients and across sites, which can imply loss of power.^36^ In order to address the problem, we implemented a recent proposal by Ewusie and colleagues called weighted segmented regression.^37,38^

Future research might benefit from a larger sample size as well as the assessment of patient-related factors such as medical comorbidities or frailty to allow for segmentation of the patient population and de-aggregation of the data to identify who benefits and why. Furthermore, a deeper understanding of the implementation process will be key such as readiness for change, leadership involvement and endorsement, staffing ratios, and availability of real-time data to support practice changes. Together, this could increase our insights into what factors contribute to the successful implementation and sustainability of delirium programs.

## CONCLUSION

Our results demonstrate that the implementation of a multicomponent delirium program on general medicine hospital units is associated with reductions in delirium and falls, with the strongest effects seen in the most vulnerable patients. Hence, we believe that multi-component interventions focused on education and screening, with delirium prevention and management strategies, can have demonstrable impacts on changing patient outcomes, reduce harm, and improve practice.

### Supplementary Information

Below is the link to the electronic supplementary material.

Supplementary file1 (DOCX 105 kb)

## Data Availability

Available upon request.

## Declarations:

### Permission to Reproduce Material from Other Sources:

NA
