# Supplementary material for: Effectiveness of a Multi-component Delirium Prevention Program Implemented on General Medicine Hospital Units: an Interrupted Time Series Analysis
Source: J Gen Intern Med. 2023 Jul 10;38(13):2936–44. doi: 10.1007/s11606-023-08238-9 (PMC10593633; doi:10.1007/s11606-023-08238-9)
Supplement: Supplementary file 1 — Supplementary file1 (DOCX 105 kb) [file 11606_2023_8238_MOESM1_ESM.docx]

**Appendix 1:** **Multi Component Delirium Program Details**

The pre-implementation activities included but were not limited to:

1. Current state mapping: a member from the quality and safety department led a co-design sessions with key stakeholders from the hospital units and the project team to map the current state and tailor the program to best fit with the flow of that unit.
2. Delirium Champions on the unit: for each unit multiple delirium champions were recruited from local allied health and nursing staff and trained in a 4 hour workshop to support the implementation and sustainability of the Delirium Program.
3. Creation of Delirium Data Dashboard: leaders of the unit would regularly receive data on the delirium screening completion rates (3 times per week), the number of positive screens (weekly) and screening trends (monthly).

After the pre-implementation activities were completed all clinical staff of the units were trained and the Delirium Program was implemented on the hospital units. The key components of the delirium program are described below and in Figure 1.

- Education: different education modalities (skills days, lunch and learn, coffee break education, webinars and seminars) were used to inform, educate and train leadership and management, unit champions, and point of care staff (nurses, occupational therapists, physical therapists, social workers and personal support workers) on delirium, delirium prevention strategies, delirium recognition including training on the use of the Confusion Assessment Method (CAM), delirium management/treatment and how to be a delirium champion. Sporting documentation was also provided in the form of posters, lanyard cards, delirium order set, and a decision making algorithm.
- Delirium prevention: The delirium prevention component focused on the use of both non-pharmacological and pharmacological strategies such as: communication, pain management, hydration, bladder and bowel functioning, mobility, eating meals in a seat, sleep hygiene, medication review and room environment.
- Delirium recognition: nursing staff on the unit screen every patient twice per day with the Confusion Assessment Method (CAM)^21^ a brief delirium diagnostic tool that is accurate (sensitivity 86%, specificity 93%), with high inter-observer reliability.^24^
- Delirium management: In cases where delirium was identified, a referral went out (automatically generated by the hospital system after two positive CAM’s were logged within 48 hours or manually by the nurse or Most Responsible Physician) to the delirium consultation team (DCT) that is comprised of an Occupational Therapist (OT), a Nurse Practitioner (NP), a Geriatrician and a Geriatric Psychiatrist. The DCT would provide a comprehensive assessment of the patient’s symptoms, function, and needs on which recommendations were made regarding for example: mobility, toileting, oral intake, environmental adaptations, communication strategies, sleep strategies, patient engagement/stimulation strategies, sensory needs, medication changes, and diagnostic investigations. Additionally, the team also provides staff, patient and family education and support.

**Appendix 2: CHART- Delirium: Chart Abstraction Tool, v. 1.4, July 14, 2016**

|  | | | | Circle Answer |
| --- | --- | --- | --- | --- |
| Study ID: | | | |  |
| 1. Describe each reference to acute confusion in the chart, verbatim (use exact words) | | | | |
| Date  (Year, Month, Date) | Time  ( : AM/PM) | | Source  (RN, MD, OT, etc.) | Description  (verbatim, in detail) |
|  |  | |  |  |
|  |  | |  |  |
|  |  | |  |  |
|  |  | |  | *Continue on another piece of paper if needed* |
| Consultations? | | Geriatrics Y N | | Date of initial consultation |
|  |  | Geriatric Psychiatry Y N | | Date of initial consultation |
|  |  | Psychiatry Y N | | Date of initial consultation |
|  |  | Occupational Therapy Y N | | Date of initial consultation |
|  |  | Physical Therapy Y N | | Date of initial consultation |
| 2. Is there any evidence in the chart of acute confusion (e.g. delirium, mental status change, disorientation, hallucinations, agitation, etc.)?  Review the entire medical record, including progress notes, nursing notes, consult notes, etc. | | | | Yes No  Uncertain |
| 3. What is the source of information about the **first** episode of acute confusion? | | | | (select from menu) ED Physician Note Admission Note  Physician’s Progress notes Nurse’s Notes Consult Note  Discharge Summary |
| 4. Approximate time of onset of first episode of acute confusion? | | | | Date: / /  Year Month Day  Or Date Uncertain |
| Check nurse’s notes, progress notes, orders, laboratories, for earliest time recorded referable to the event. | | | | Time: : AM / PM Or Time Uncertain |
| 5. What was the total duration (in days) of confusion?  i.e. as determined by all references to confusion in the chart | | | | days uncertain |
| 6. Was there any evidence of agitation associated with the delirium?  i.e. hyperactive delirium | | | | Yes, No, Uncertain  If Yes or Uncertain, Describe: |
| 7. Was there any evidence of reversibility or improvement of acute confusion during the hospitalization? | | | | Yes, No, Uncertain  If Yes or Uncertain, Describe: |
| 8. Was a cognitive screening tool performed? | | | | Yes/No |
| 9. If Cognitive Screening Tool performed, document scores | | | | Date: |
| for all tests: | | | | Test (pick which one): |
|  | | | | MMSE/MOCA |
|  | | | | Total Score: |
|  | | | | Notes (if any comments, for |
|  | | | | example, patient only |
|  | | | | completed half the |
|  | | | | test): |
| **Delirium Present?** | | | | Yes No  Uncertain |


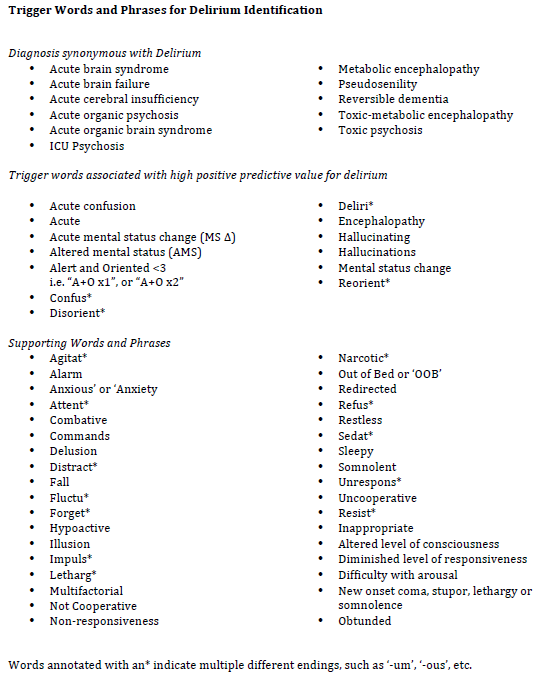


**Appendix 3:** **Segmented regression models**

Segmented regression models fit a least squares regression line to each segment of the independent variable, time, and thus assume a linear relationship between time and the outcome within each segment. We can specify the following linear regression model to estimate the level and trend in number of delirium cases (or falls) before the intervention and the changes in level and trend following the intervention:

Y_t_=β_0_+β_1_*time_t_+β_2_*intervention_t_+β_3_*time after intervention_t_+*e*_t_

where Y_t_ is the number of delirium cases (or falls) in month t; time is a continuous variable indicating time in months at time t from the start of the observation period; intervention is an indicator for time t occurring before (intervention = 0) or after (intervention = 1) the intervention, which was implemented at month 9 in the series; and time after intervention is a continuous variable counting the number of months after the intervention at time t, coded 0 before the intervention and (time – 8) after the intervention.

In this model, β_0_ estimates the baseline level of the outcome, number of delirium cases (or falls) per month, at time zero; β_1_ estimates the change in the number of delirium cases (or falls) that occurs with each month before the intervention (i.e. the baseline trend); β_2_ estimates the level change in the monthly number of delirium cases (or falls) immediately after the intervention, that is, from the end of the preceding segment; and β_3_ estimates the change in the trend in the monthly number of delirium cases (or falls) after the intervention, compared with the monthly trend before the intervention. The sum of β_1_ and β_3_ is the post-intervention slope. The error term *e*_t_ at time t represents the random variability not explained by the model. It consists of a normally distributed random error and an error term at time t that may be correlated to errors at preceding or subsequent time points.

Tables 1a and 1b contain the parameter estimates of the linear segmented regression model specified above for the monthly number of delirium cases and falls, respectively. After adjusting the models for the negative autocorrelation in the error term with the Cochrane-Orcutt procedure, the results do not change significantly in both models. However, after accounting for the heterogeneity across units, the effect of the multicomponent delirium intervention on the monthly number of delirium cases slightly reduced, whereas the models for the number of falls were not significant.

Table 1a. Parameter estimates, standard errors and P-values from the full and most parsimonious segmented regression models predicting monthly number of delirium cases.

|  | Coefficient | Standard Error | t-statistic | P-value |
| --- | --- | --- | --- | --- |
| a. Full segmented regression model | | | | |
| Intercept β_0_ | 10.4 | 1.86 | 5.58 | 0.00 |
| Baseline trend β_1_ | -0.06 | 0.37 | -0.16 | 0.88 |
| Level change after intervention β_2_ | -2.49 | 2.42 | -1.03 | 0.32 |
| Trend change after intervention β_3_ | 0.05 | 0.52 | 0.09 | 0.93 |
| b. Most parsimonious segmented regression model* | | | | |
| Intercept β_0_ | 10.13 | 0.78 | 12.92 | 0.00 |
| Level change after intervention β_2_ | -2.75 | 1.11 | -2.48 | 0.03 |
| c. Most parsimonious segmented regression model (Cochrane-Orcutt estimation)** | | | | |
| Intercept β_0_ | 10.16 | 0.55 | 18.63 | 0.00 |
| Level change after intervention β_2_ | -2.71 | 0.77 | -3.5 | 0.04 |
| d. Most parsimonious weighted segmented regression model^§^ | | | | |
| Intercept β_0_ | 9.559 | .791 | 12.085 | .000 |
| Level change after intervention β_2_ | -2.472 | .999 | -2.473 | .027 |
| e. Most parsimonious weighted segmented regression model^§§^ | | | | |
| Intercept β_0_ | 9.495 | .826 | 11.499 | .000 |
| Level change after intervention β_2_ | -2.355 | 1.021 | -2.305 | .037 |

*Durbin-Watson 2.88; **Durbin-Watson 2.12

^§^For each time t, the weight is 1/variance between units.

^§§^ For each time t, the weight is 1/weighted variance between units (weighted by unit sample size).

Table 1b. Parameter estimates, standard errors and P-values from the full and most parsimonious segmented regression models predicting monthly number of falls.

|  | Coefficient | Standard Error | t-statistic | P-value |
| --- | --- | --- | --- | --- |
| a. Full segmented regression model | | | | |
| Intercept β_0_ | 14.5 | 2.12 | 6.83 | 0.00 |
| Baseline trend β_1_ | 6.723E-17 | 0.42 | 0.00 | 1.00 |
| Level change after intervention β_2_ | -1.68 | 2.76 | -0.6 | 0.55 |
| Trend change after intervention β_3_ | -0.24 | 0.6 | -0.4 | 0.7 |
| b. Most parsimonious segmented regression model* | | | | |
| Intercept β_0_ | 14.5 | 0.9 | 16.04 | 0.00 |
| Level change after intervention β_2_ | -2.75 | 1.28 | -2.15 | 0.049 |
| c. Most parsimonious segmented regression model (Cochrane-Orcutt estimation)** | | | | |
| Intercept β_0_ | 14.87 | 0.6 | 24.5 | 0.00 |
| Level change after intervention β_2_ | -3.09 | 0.86 | -3.6 | 0.004 |
| d. Most parsimonious weighted segmented regression model^§^ | | | | |
| Intercept β_0_ | 12.542 | .933 | 13.445 | .000 |
| Level change after intervention β_2_ | -1.064 | 1.260 | -.845 | .412 |
| e. Most parsimonious weighted segmented regression model^§§^ | | | | |
| Intercept β_0_ | 12.193 | .876 | 13.918 | .000 |
| Level change after intervention β_2_ | -.802 | 1.271 | -.631 | .538 |

*Durbin-Watson 2.64;**Durbin-Watson 2.12.

^§^For each time t, the weight is 1/variance between units.

^§§^ For each time t, the weight is 1/weighted variance between units (weighted by unit sample size).
